# Resistance of Black Aspergilli Species from Grape Vineyards to SDHI, QoI, DMI, and Phenylpyrrole Fungicides

**DOI:** 10.3390/jof9020221

**Published:** 2023-02-07

**Authors:** Stefanos I. Testempasis, George S. Karaoglanidis

**Affiliations:** Laboratory of Plant Pathology, School of Agriculture, Aristotle University of Thessaloniki, P.O. Box 269, 54124 Thessaloniki, Greece

**Keywords:** *Aspergillus uvarum*, *Aspergillus tubingensis*, pyraclostrobin, fluxapyroxad, *Vitis vinifera*

## Abstract

Fungicide applications constitute a management practice that reduces the size of fungal populations and by acting as a genetic drift factor, may affect pathogen evolution. In a previous study, we showed that the farming system influenced the population structure of the *Aspergillus* section *Nigri* species in Greek vineyards. The current study aimed to test the hypothesis that the differences in the population structure may be associated with the selection of fungicide-resistant strains within the black aspergilli populations. To achieve this, we determined the sensitivity of 102, 151, 19, and 22 for the *A. uvarum*, *A. tubingensis*, *A. niger*, and *A. carbonarious* isolates, respectively, originating either from conventionally-treated or organic vineyards to the fungicides fluxapyroxad-SDHIs, pyraclostrobin-QoIs, tebuconazole-DMIs, and fludioxonil-phenylpyrroles. The results showed widespread resistance to all four fungicides tested in the *A. uvarum* isolates originating mostly from conventional vineyards. In contrast, all the *A. tubingensis* isolates tested were sensitive to pyraclostrobin, while moderate frequencies of only lowly resistant isolates were identified for tebuconazole, fludioxonil, and fluxapyroxad. Sequencing analysis of the corresponding fungicide target encoding genes revealed the presence of H270Y, H65Q/S66P, and G143A mutations in the *sdhB*, *sdhD*, and *cytb* genes of *A. uvarum* resistant isolates, respectively. No mutations in the *Cyp51A* and *Cyp51B* genes were detected in either the *A. uvarum* or *A. tubingensis* isolates exhibiting high or low resistance levels to DMIs, suggesting that other resistance mechanisms are responsible for the observed phenotype. Our results support the initial hypothesis for the contribution of fungicide resistance in the black aspergilli population structure in conventional and organic vineyards, while this is the first report of *A. uvarum* resistance to SDHIs and the first documentation of H270Y or H65Q/S66P mutations in *sdhB*, *sdhD*, and of the G143A mutation in the *cytb* gene of this fungal species.

## 1. Introduction

Grape vine yield and the quality of grape products is affected by a long series of biotic and abiotic factors. Among them, pathogens affecting berries as either pre- or postharvest spoilage agents are the most important. Aspergillus species belonging to *Aspergillus* section *Nigri*, known also as black aspergilli, are among the most important pre-or postharvest spoilage agents causing a disease known as Aspergillus rot [1]. The presence of black aspergilli has been reported throughout the world, particularly in vineyards grown under hot and dry conditions [2,3,4].

Currently, *Aspergillus* section *Nigri* includes, in total, 30 species that have been isolated from grapes and among them, the biseriate *A. carbonarius*, *A. niger*, *A. tubingensis*, *A. brasieliensis* and the uniseriate *A. uvarum*, *A. japonicus*, and *A. aculeatus* are the most important [5,6,7,8]. Among these species, of great concern are those producing mycotoxins, with ochratoxin A (OTA) and fumonisins (FB1, FB2, and FB4) being the most common in wines, raisins, and other grape products. OTA is produced by a species of *Aspergillus* section *Circumdati* and *Aspergillus* section *Nigri* group, with *A. carbonarious* being the main OTA producer on grapes and grape products, while strains of *A. niger* can also produce the same mycotoxin, but to a lower extent [9]. In grapes, fumonisin contamination has been associated with *A. niger* [10]. In addition to the quantitative or qualitative losses to grape yield, some of the above-mentioned *Aspergillus* section *Nigri* species such as *A. niger*, *A. tubingensis*, or *A. uvarum* are associated with pulmonary aspergillosis and otomycosis in humans or domestic and wild animals [11,12,13].

Efforts to reduce the risk for *Aspergillus* infections on grapes and the mycotoxin contamination of grape products have focused on attempts to prevent fungal growth on grapes, since berry infection by these opportunistic pathogens occur in the vineyard from veraison to harvest. This has been recognized as the most effective tactic, and several natural fungicides (i.e., fusopyrone, perillaldehyde, natamycin) or biological control agents (i.e., *Aureobasidium pullullans*, *Candida* spp., *Lanchacea thermotolerans*) have been evaluated both in vitro and in vineyards against *Aspergillus* spp. [9,14,15]. However, conventional fungicides have been evaluated and used as the main tool to prevent the growth of black aspergilli on grapes. Fungicides containing the phenylpyrrole derivative fludioxonil have been shown to be the most effective in reducing the black aspergilli incidence on grapes [1,16,17], while members of other fungicide groups such as quinone outside inhibitors (QoIs), sterol demethylation inhibitors (DMIs), or anilinopyrimidines may reduce *Aspergillus* spp. incidence and mycotoxin production at lower rates [17].

Throughout the world, vineyards represent agroecosystems heavily treated with fungicides that are applied to protect vines and berries from several important foliar or fruit diseases. Major target site-specific fungicide groups registered for use in Greek vineyards against grape diseases include sterol demethylation inhibitors (DMIs), (flutriafol, difenoconazole, tebuconazole, tetraconazole, myclobutanil), quinone outside inhibitors (QoIs), (azoxystrobin, trifloxystrobin, pyraclostrobin), succinate dehydrogenase inhibitors (SDHIs), (boscalid, fluopyram, fluxapyroxad), anilinopyrimidines (pyrimethanil, cyprodinil), and phenylpyrroles (fludioxonil). Most of these fungicides are used against diseases such as powdery mildew, downy mildew, or Botrytis bunch rot. However, although they are not used primarily against black aspergilli, the fungal populations may be exposed to selection pressure even as non-targets of the applications. However, this intensive fungicide use imposes the risk of resistance development by pathogens exposed to the selection pressure in the vineyard environment [18,19,20].

Until recently, fungicide resistance studies in black aspergilli have mainly focused on species of medical importance such as *A. fumigatus*. It is a saprophytic fungus living in the soil but its airborne spores may infect humans, causing chronic pulmonary aspergillosis or invasive aspergillosis [21]. The development of resistance by this fungal species to azole (DMIs) fungicides is a major concern and for several years, it has been recognized that the selection of resistant strains may occur due to fungal exposure to agricultural DMIs applied against plant pathogens [22,23]. In contrast to *A. fumigatus*, reports on fungicide resistance development by black aspergilli associated with Aspergillus rot of grape are sparse. Only recently were the first cases of resistant selection reported in the U.S. by Cosseboom and Hu [24], who provided evidence for the selection of F129L mutants conferring resistance to QoIs in *A. uvarum* isolated from grapes.

In a recent study aiming to determine the population structure of *Aspergillus* section *Nigri* species, it was found that the farming system (conventional vs. organic vineyards) may significantly affect both the frequency of *Aspergillus* presence on the bunches and the fungal composition at the species level [25]. In detail, our experimental data showed that in conventionally-treated vineyards, the predominant *Aspergillus* section *Nigri* species was *A. uvarum*, while in organic vineyards, *A. tubingensis* dominated the population. Factors contributing to those differences in population structure in vineyards cultivated with different cropping systems remain largely unknown. Therefore, this study was initiated to test the hypothesis that differences in the frequency of black aspergilli on grape bunches and population structure may be due to the selection of fungicide-resistant strains in conventionally treated vineyards. To test this hypothesis, the sensitivity of *A. uvarum*, *A. tubingensis*, *A. niger*, and *A. carbonarious* isolated from organic and conventionally-treated vineyards to the DMI tebuconazole, the SDHI fluxapyroxad, the QoI pyraclostrobin, and the phenylpyrrole fludioxnil was measured. In the course of the study, moderate to high frequencies of resistance to SDHIs and QoIs were observed within the *A. uvarum* fraction of the population tested and for these fungicide classes, the molecular mechanisms of resistance were investigated in the resistant isolates by sequencing the respective target genes.

## 2. Materials and Methods

### 2.1. Fungal Isolates

A total of 102, 151, 19, and 22 isolates of *A. uvarum*, *A.tubingensis*, *A. niger*, and *A. carbonarius*, respectively, were tested in this study. The isolates were collected from vineyards throughout Greece for the requirements of a study aiming to explore the impact of the cropping system (conventional vs. organic) on the incidence of black aspergilli species and the associated population structure [25]. The identification of the collected isolates of *Aspergillus* section *Nigri* species was performed by the sequence analysis of three (3) reference genes (*ITS*, *β-tubulin*, and *calmodulin*), [25]. The isolates were maintained on acidified potato dextrose agar (PDA, Merck, Darmstadt, Germany) at 4 °C until use. Detailed information on the number of isolates per species and cropping system is provided in Table S1.

### 2.2. Fungicides

Fungicides used in the study were selected among the main groups of single-site inhibitors registered for use in Greek vineyards against foliar or bunch diseases. The commercial formulations of fludioxonil (Geoxe 50 WG, Syngenta, Basel, Switzerland), fluxapyroxad (Sercadis 30 SC, BASF, Ludwigshafen, Germany), fluopyram (Luna Privilege 50 SC, Bayer CropScience, Leverkusen, Germany), boscalid (Cantus 50 WG, BASF, Ludwigshafen, Germany), pyraclostrobin (Insignia 20 WG, BASF, Ludwigshafen, Germany), isofetamid (Kenja 40 SC, Agrology Papaoikonomou, Thessaloniki, Greece), penthiopyrad (Fontelis 20 SC, Corteva AgriScience, Indianapolis, IN, USA), and tebuconazole (Folicur 25 WG, Bayer CropScience, Leverkusen, Germany) were used in the sensitivity assays.

### 2.3. Fungicide Sensitivity Measurements

The sensitivity of the collected isolates of each Aspergillus species (*A. uvarum*, *A. tubingensis*, *A. carbonarius*, and *A. niger*) to the selected fungicides was determined using a range of different concentrations. Details on the concentrations of fluxapyroxad, fludioxonil, pyraclostrobin, and tebuconazole are provided in Table S2.

Fungicide sensitivity assessments were based on the measurement of mycelial growth inhibition for fluxapyroxad, fludioxonil, and tebuconazole or conidia germination inhibition for pyraclostrobin. In the study, different culture media were used to determine the fungicide sensitivity since the growth media should promote both fungal growth and fungicide activity. To determine sensitivities to fludioxonil, tebuconazole, and fluxapyroxad, conidia were harvested by sterile cotton swabs from three-day-old culture of each isolate and transferred into an Eppendorf tube (1.5 mL) containing 1 mL of sterile distilled water to re-suspend the conidia. Afterward, the spore suspension was incorporated into a 90 mm Petri dish with 15 mL of PDA and mixed thoroughly. Cultures were incubated in a growth chamber for 48–72 h (27 °C, dark). Then, mycelial plugs were transferred using a 6 mm core borer onto Roswell Park Memorial Institute-1640 (RPMI-1640) agar plates (RPMI-1640 Agar with MOPS and two glucose/without sodium bicarbonate, Himedia, Mumbai, India), amended with a range of fluxapyroxad or fludioxonil concentrations or on PDA plates amended with several concentrations of tebuconazole (Table S2). The cultures were incubated at 27 °C in the dark for three (3) days, and then, the diameter of the colonies was measured. Sensitivities to pyraclostrobin were estimated by plating aliquots (30 μL) of the conidia suspension (1 × 10^4^ conidia mL^−1^) of each isolate with a sterile colony spreader on a 2% water agar (WA) medium amended with several concentrations of pyraclostrobin (Table S2) and 100 mg L^−1^ of salihydroxamic acid (SHAM, Sigma Co., Saint Louis, MO, USA) to inhibit the alternative oxidation pathway. The conidia were incubated for 18 h at 27 °C in the dark, and then the number of germinated conidia was measured microscopically. A conidium was considered as germinated when its length was twice the length of the condium. Each isolate was tested in triplicate, while the control cultures for each fungal isolate were cultivated on the respective media (PDA, WA, and RPMI-1640 agar) without any fungicide.

### 2.4. DNA Extraction

Mycelia plugs from the three day-old cultures were transferred into 100 mL culture flasks containing 30 mL potato dextrose broth (PDB, Neogen, Scotland, UK). Cultures were incubated for 7 days (25 °C, dark), and the produced mycelia were harvested by filtration and lyophilized. DNA was extracted with the QIAamp DNA Mini Kit (Qiagen GmbH, Hilden, Germany) according to the manufacturer’s protocol.

### 2.5. Fungicide Target Gene Amplification Associated with Resistance to SDHIs, QoIs, and DMIs

To investigate the presence of target site mutations associated with resistance to the tested fungicides, the *sdhB*, *sdhC*, *sdhD*, *Cyp51A*, *Cyp51B*, and *cytb* genes of the *A. uvarum* and *A. tubingensis* isolates were amplified and sequenced. Primers were designed based on published sequences of the targeted genes of *A. uvarum* and *A. tubingensis* strains or other *Aspergillus* species (Table S3). The sequences were mapped to the reference genomes of *A. uvarum* (Genbank Accession GCA_003184745.1) and *A. tubingensis* (Genbank Accession GCA_001890745), while primer pairs were generated using the Geneious Prime^®^ 2022.1.1 (Biomatters Ltd., Auckland, New Zealand) software.

PCR reactions were carried out using the polymerase KAPA HiFi HotStart ReadyMix (Roche Sequencing Store Inc., Indianapolis, IN, USA). Reactions were performed in a final volume of 25 μL containing 12.5 μL 2X KAPA HiFi HotStart ReadyMix buffer, 8 μL of molecular biology grade water (PanReac AppliChem, Darmstadt, Germany), 3 μL of genomic DNA (10 ng/μL), and 0.75 μL of each primer (10 μM primer stocks). The PCR cycling protocol used was as follows: initial denaturation at 95 °C for 3 min, followed by 35 cycles of denaturation (98 °C for 20 s), annealing (15 s at the respective annealing temperature), and elongation (1 or 2 min at 72 °C; depending on the amplicon’s size). The final cycle was followed by an extension (1 min at 72 °C) and a hold step at 4 °C. Detailed information on the primer’s sequence, annealing temperatures, and amplicon sizes are presented in Table 1. PCR product purification was performed using the Monarch PCR and DNA Clean-Up Kit (New England Biolabs, Ipswich, MA, USA) following the manufacturer’s instructions, while Sanger sequencing analysis was conducted at Azenta/GENEWIZ (Azenta Life Sciences, Waltham, MA, USA). Sequences were processed and aligned using the Geneious Prime^®®^ 2022.1.1 (Biomatters Ltd., Auckland, New Zealand) software.

### 2.6. Cross-Resistance Relationships between SDHI Fungicides

The sensitivity of the isolates with single or/and double sdh mutations to several SDHIs (fluxapyroxad, boscalid, penthiopyrad, isofetamid, and fluopyram) was tested by measuring the inhibition of the spores’ germ tube length. In total, four (4) isolates (A137, A127, B7, and B9) with the H270Y mutation in the *sdh*B subunit, five (5) isolates (B14, A67, A69, A121, and B144) possessing the H65Q/P66S mutations in the *sdh*D subunit, three (3) isolates (B10, B11, and B12), possessing all three *sdh* mutations, and three (3) isolates that were sensitive to all of the tested fungicides were included in the study. The fungicide concentrations used were 0, 0.01, 0.05, 0.1, 0.5, 1, 5, 10, and 50 mg L^−1^. Aliquots (30 μL) of the conidia suspension of each isolate (1 × 10^4^ conidia mL^−1^) were plated with a sterile colony spreader on RPMI-1640 agar medium amended or not with each fungicide concentration. Cultures were incubated for 18 h in a growth chamber (27 °C, dark) and after the end of the incubation period, the length of the germination tube was measured in 50 conidia per isolate and fungicide concentration.

### 2.7. Data Analysis

The effective concentration at which mycelial growth, conidia germination, and the length of the germination tube was inhibited by 50% (EC50) was calculated by plotting the relative inhibition against the Log10 of fungicide concentrations. EC50 values were calculated using GraphPad Prism, version 9.4.0 software (GraphPad Software, San Diego, CA, USA), while the resistance factors (RFs) were estimated by dividing the EC50 value for each isolate by the mean EC50 values of the most sensitive isolates. The RF values were used to characterize the resistance phenotype. In particular, isolates with RF < 2 were classified as sensitive (S), whereas isolates with RF > 2 were assigned as low resistant (LR). Highly resistant (HR) isolates were those whose growth was not inhibited, even at the highest tested dosages.

## 3. Results

### 3.1. Fungicide Sensitivity of Different Black Aspergilli Species

Sensitivity measurements to the selected fungicides revealed that the four black aspergilli species tested were considerably differentiated in their fungicide sensitivity profile (Table 2). *A. uvarum* and *A. tubingensis* isolates showed a lower sensitivity to most of the fungicides compared to that of *A. niger* and *A. carbonarius*. In *A. uvarum*, highly resistant (HR) isolates were detected against all four fungicides tested. The higher frequencies of highly resistant isolates were detected against fluxapyroxad and pyraclostrobin (17 and 16 out of 102 *A. uvarum* isolates, respectively) (Table 2). In contrast, the number of *A. uvarum* isolates that were highly resistant to tebuconazole or fludioxonil was lower (six and two isolates out of 102, respectively). Furthermore, a relatively high fraction of the *A. uvarum* isolates was of low resistance (LR) to fluxapyroxad (33 out of 102 isolates) and to fludioxonil (44 out of 102 isolates). In *A. tubingensis*, high resistance was detected only against fludioxonil in the limited number of four out of 151 isolates, while all of them were sensitive to pyraclostrobin. However, a significant fraction of the tested *A. tubingensis* population was found to be of low resistance to fluxapyroxad, tebuconazole, and fludioxonil with 31, 58, and 22 isolates out of 151, respectively.

The entire set of *A. niger* isolates was highly sensitive to fluxapyroxad, pyraclostrobin, and fludioxonil with RF values lower than 2, while for tebuconazole, only a small fraction of the *A. niger* isolates showed low resistance levels with RF values ranging from 2.1 to 5.3, with the remaining being highly sensitive (Table 2). Similarly, the entire set of *A. carbonariοus* isolates tested was highly sensitive to pyraclostrobin, with RF values ranging from 0 to 1.2. In contrast, all 22 *A. carbonarious* isolates tested were found to be insensitive to fluxapyroxad, since no inhibition of mycelial growth was observed, even at the highest concentration tested of 20 μg mL^−1^ in any of the isolates. Even though most of the *A. carbonarious* isolates were sensitive to tebuconazole (*n* = 14) and fludioxonil (*n* = 20), few LR isolates were identified with RF values ranging from 2.2 to 5.1 and from 2 to 4.5, respectively (Table 2).

### 3.2. Frequencies of S, LR, and HR Isolates Based on the Farming System

**Figure 1:**
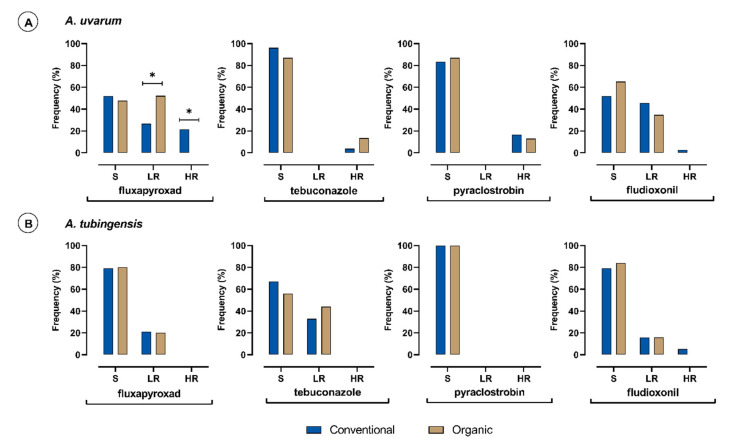
Frequency (%) of sensitive (S), lowly resistant (LR), and highly resistant (HR) isolates of *Aspergillus uvarum* (**A**) (*n* = 102) and *Aspergillus tubingensis* (**B**) (*n* = 151) populations obtained from conventional (blue columns) or organic (brown columns) vineyards to fluxapyroxad, tebuconazole, pyraclostrobin, and fludioxonil. Asterisk (*) indicates significant differences between the two farming systems according to a series of *z*-test analyses (*p* = 0.05).

The frequencies of sensitive (S), lowly resistant (LR), and highly resistant (HR) isolates of *A. uvarum* and *A. tubingensis* species to each fungicide tested were calculated based on their farming system of origin (Figure 1). Within the *A. uvarum* subpopulation, the number of isolates originating from conventional vineyards (*n* = 79) or organic vineyards (*n* = 23) was uneven, since *A. uvarum* isolated in low frequencies from organic vineyards. In contrast, the numbers of *A. tubingensis* isolates originating from either conventional (*n* = 76) or organic vineyards (*n* = 75) were equal. Isolates of *A. carbonarious* and *A. niger* were not included in this comparison due to the limited number of isolates obtained. Marked differences were observed in the frequencies of isolates of *A. uvarum* with different sensitivity levels to fluxapyroxad, based on their origin. All of the highly fluxapyroxad-resistant *A. uvarum* isolates (*n* = 17) originated from conventional vineyards (Figure 1A). In contrast, no differences were observed in the frequency of fluxapyroxad lowly resistant isolates of *A. tubingensis*, which was detected in the frequency of 21% within each group (Figure 1B). Regarding pyraclostrobin, highly resistant isolates (*n* = 16) were detected only in *A. uvarum*, while the *A. tubingensis* subpopulation was highly sensitive. Thirteen out of 16 *A. uvarum* highly resistant isolates originated from conventional vineyards, while only three originated from organic vineyards. Their frequencies within each subpopulation were not significantly different because of the uneven sample size (Figure 1A). Isolates highly resistant to fludioxonil were detected in both *A. uvarum* and *A. tubingensis* subpopulations originating only from the conventional vineyards, but their number was too low (two and four isolates, respectively). In the *A. tubingensis* subpopulation, isolates with a low resistance to fludioxonil were observed in balanced frequencies in the conventionally and organically treated vineyards with a value of 15.5% (Figure 1B). Isolates with low resistance levels to fludioxonil were also detected in the *A. uvarum* subpopulation, but in this case, the frequency of low resistant isolates originating from conventionally-treated vineyards was significantly higher compared to that in the organic vineyards (Figure 1A). Regarding tebuconazole, highly resistant isolates were only detected in the *A. uvarum* subpopulation but in very low numbers, evenly split from the conventionally treated and organic vineyards. In the *A. tubingensis* subpopulation, only isolates with low resistance levels to tebuconazole were detected, and their frequencies within the subpopulations originating from either conventionally-treated or organic vineyards were similar (Figure 1B).

### 3.3. Molecular Characterization of SDHI-, QoI- and DMI-Resistant Isolates

**Figure 2:**
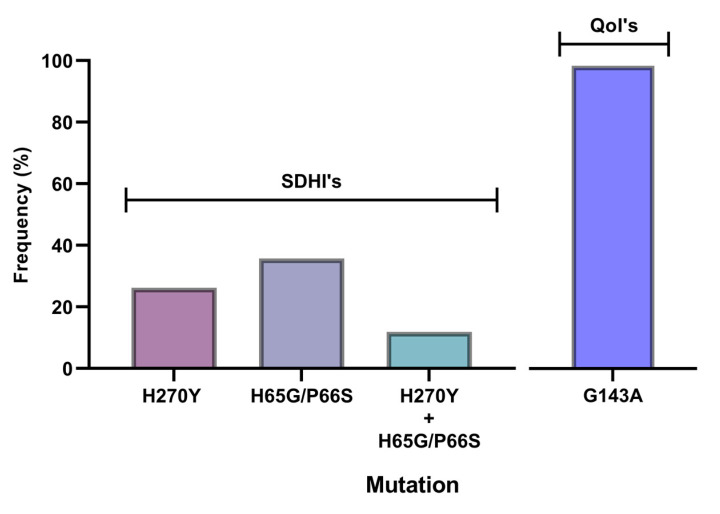
Frequency (%) of H270Y, H65Q/P66S, H270Y, and H65Q/P66S, and the G143A mutants of the *Aspergillus uvarum* population within the fluxapyroxad-resistant (*n* = 42) and pyraclostrobin-resistant (*n* = 16) fraction of the population.

**Figure 3:**
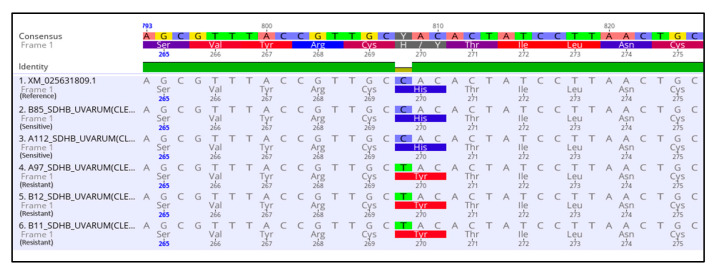
Multiple sequence alignment of nucleotides and amino acids of the *sdhB* gene in SDHI resistant isolates of *Aspergillus uvarum* possessing the H270Y mutation.

**Figure 4:**
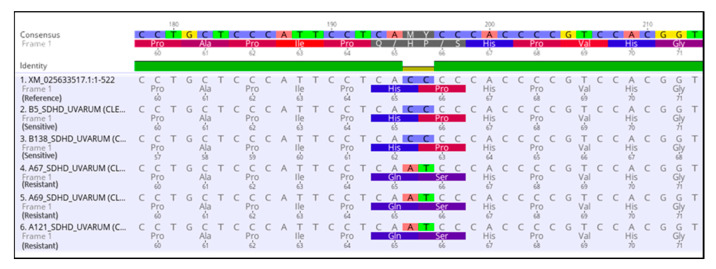
Multiple sequence alignment of nucleotides and amino acids of the *sdhD* gene in SDHI resistant isolates of *Aspergillus uvarum* possessing H65Q/P66S mutations.

To determine whether the observed resistance phenotypes were associated with the presence of mutations in the fungicide target genes, *sdhB*, *sdhC*, *sdhD*, *Cyp51*, and *cytb* genes of *A. uvarum*, *sdhB*, *sdhC*, *sdhD,* and *Cyp51* of *A. tubingensis,* and *sdhB*, *sdhC,* and *sdhD* of *A. carbonariou*s were amplified and sequenced. The sequencing of *sdhB*, *sdhC,* and *sdhD* encoding genes in *A. uvarum* revealed that 11 isolates (26.2 %) possessed a histidine (CAC) replacement by tyrosine (TAC) at codon 270 of *sdhB* (Figure 2 and Figure 3). Furthermore, in 15 (35.7%) *A. uvarum* isolates, a double mutation (H65Q/S66P) was detected in the *sdhD* subunit (Figure 2). The H65Q/S66P mutations are caused by the replacement of histidine (CAC) by glutamine (CAA) at codon 65 and of proline (CCC) by serine (TCC) at codon 66 of *sdhD* (Figure 4). Interestingly, in a limited number (*n* = 5, 11.9%) of the sequenced isolates, the identified mutations H270Υ in *sdhB* and H65Q/S66P in *sdhD* co-occurred (Figure 2). No mutation was detected in the *sdhB*, *sdhC*, and *sdhD* subunits in any of the 31 *A. tubingensis* isolates showing low levels of resistance to fluxapyroxad. Similarly, no mutation in any of the three (3) *sdh* subunits sequenced was observed in the *A. carbonarious* isolates that were found to be resistant to fluxapyroxad.

**Figure 5:**
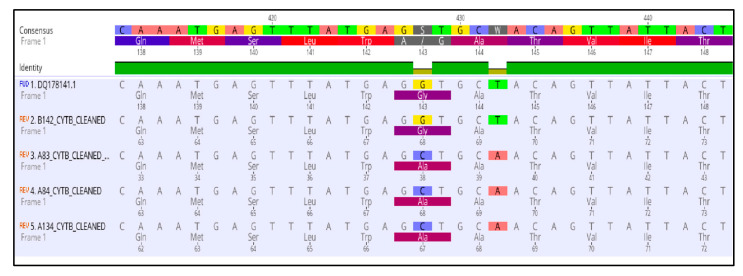
Multiple sequence alignment of the nucleotides and amino acids of the *cytochrome b* gene in the QoI resistant isolates of *Aspergillus uvarum* possessing the G143A mutation.

The measurements of sensitivity to the QoI pyrasclostrobin revealed the presence of a resistant population fraction only in *A. uvarum*, while in all three (3) remaining fungal species tested, all of the isolates were highly sensitive. Sequencing analysis of the cytb gene in the QoI-resistant *A. uvarum* isolates showed the presence of a mutation leading to the substitution of glycine (GGT) to alanine (GCT) at codon 143 (G143A) (Figure 5). All of the *A. uvarum* QoI-resistant isolates (*n* = 16, 100%) possessed this mutation (Figure 2). Albeit several LR and HR strains were identified in the populations of all four (4) *Aspergillus* spp. tested, sequencing analysis of the *Cyp51A* and *Cyp51B* encoding genes revealed the absence of any target mutation in the resistant strains. The sequences of the *sdhB*, sdhD, and cytb genes of the SDHI- or QoI-resistant isolates of *A. uvarum* possessing the H270Y, H65Q/P66S, or G143A mutations have been submitted to the National Center of Biotechnology Information (NCBI) database and the respective accession numbers are presented in Table S4.

### 3.4. Cross-Resistance Relationships between SDHIs

**Figure 6:**
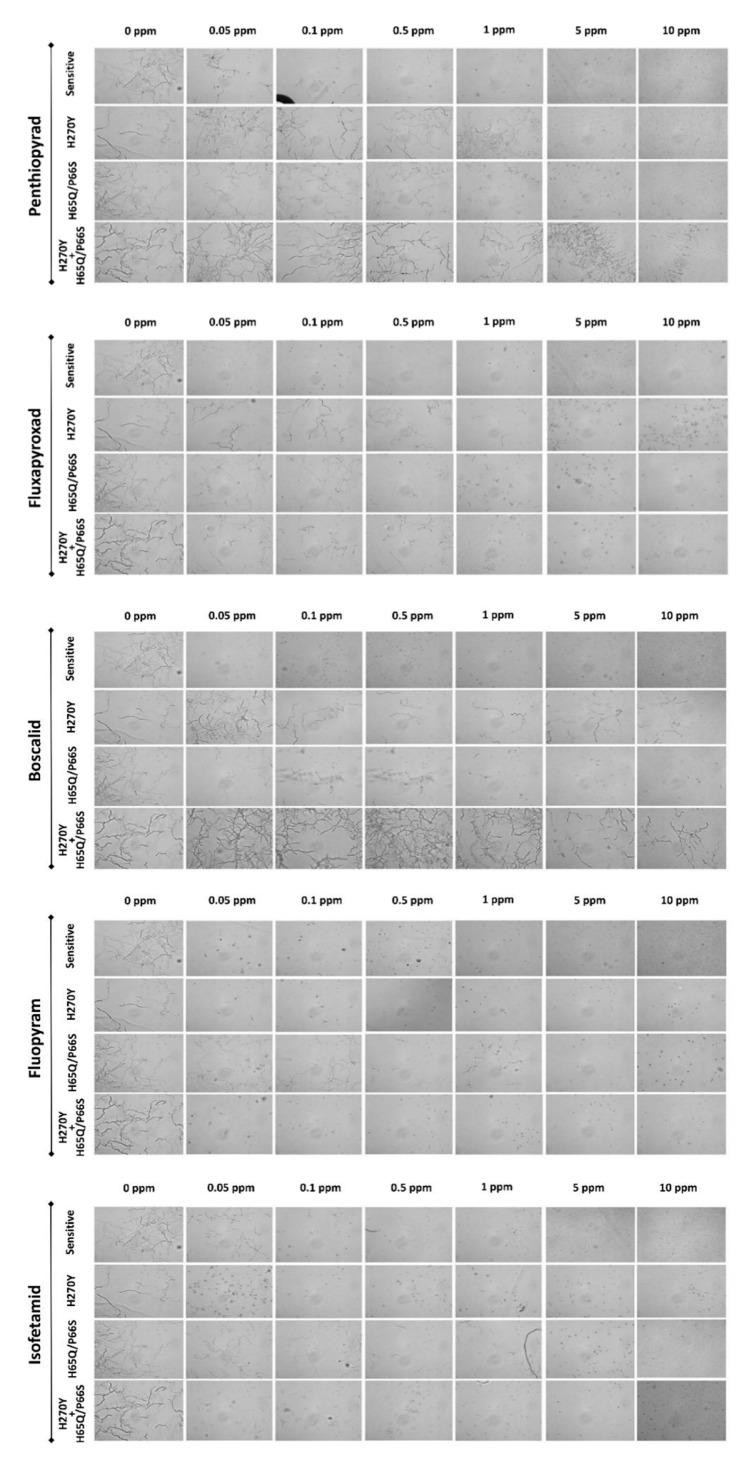
Germ tube growth of the *Aspergillus uvarum* isolates (A60-sensitive; A137—H270Y; A121-H65Q/P66S; B12—H270Y+H65Q/P66S) grown on nutrient media amended with several concentrations of the SDHI fungicides boscalid, fluopyram, isofetamid, penthiopyrad, and fluxapyroxad.

The sensitivity data of several SDHI molecules used in vineyards against fungal pathogens in the mutated and wild-type *A. uvarum* isolates are summarized in Table 3, while the response of the conidia of representative isolates, grown on different fungicide concentrations, is shown in Figure 6. Results showed a slight variation regarding the intrinsic activity of the fungicides tested against the *A. uvarum* isolates of wild-type sensitivity, with mean EC50 values ranging from 0.003 μg mL^−1^ for fluxapyroxad, which was the most active molecule, to 0.019 μg mL^−1^ for penthiopyrad, which was the least active among the five (5) SDHI molecules included in the study.

All of the isolates possessing the H270Y mutation were either moderately- or highly-resistant to fluxapyroxad, boscalid, and penthiopyrad, with RF values ranging from 50.5 to 208, 195 to 298, and 20.7 to 174, respectively. In contrast, none of the H270Y mutants were resistant to either isofetamid or fluopyram. The double mutation identified in the *sdhD* subunit (H65Q/P66S) was associated with only low resistance levels to all five SDHI molecules tested with RF values ranging from 5–15.3, 11.8–44.1, 2.7–5.7, 3.7–28.1 to 14.2–55.3 for fluxapyroxad, boscalid, penthiopyrad, isofetamid and fluopyram, respectively. The isolates possessing both the H270Y mutation in *sdhB* and the double substitution H65Q/P66S in *sdhD* had a sensitivity phenotype similar to that described previously for the H270Y mutants, exhibiting moderate to high resistance to fluxapyroxad, boscalid, and penthiopyrad while they were highly sensitive to isofetamid and fluopyram.

## 4. Discussion

This study was initiated primarily to investigate whether the differences in the frequency of the presence of black aspergilli and species composition between conventionally-treated and organic vineyards are related to the possible development of resistance to chemical fungicides used in vineyards. To achieve this, the sensitivity of a large set of black aspergilli isolates belonging to several species and originating from either conventionally- or organically-treated vineyards was measured by calculating the EC50 values after exposure to several commonly used fungicides in vineyards. Vineyards represent, throughout the world, an environment heavily treated with fungicides applied to combat several foliar and bunch diseases [26]. Even though there are numerous studies aiming to determine the fungicide sensitivity profile of important grapevine pathogens such as *Plasmopara viticola*, *Erysiphe necator*, or *Botrytis cinerea* [18,21], the information regarding the fungicide sensitivity status of black aspergilli populations originating from vineyards is very limited [27]. Therefore, this study represents the first report on the fungicide sensitivity levels of major black aspergilli species associated with the Aspergillus bunch rot of grapes.

In a previous study by our group, it was found that in conventionally-treated vineyards, the incidence of black aspergilli at harvest was higher compared to that in organic vineyards located in the same region and cultivated with the same varieties as conventionally-treated vineyards [25]. Furthermore, in the conventionally treated vineyards, the predominant *Aspergillus* species was *A. uvarum*, while in the respective organic vineyards, *A. tubingensi*s was found to be the predominant species [25]. In accordance with these findings, previous reports showed a lower incidence of black aspergilli in organic vineyards compared to those conventionally treated [28], or a lower risk for mycotoxin contamination in wines originating from organic vineyards compared to those from conventionally-treated vineyards [29]. Interestingly, similar findings of higher frequencies of mycotoxigenic *Fusarium* spp. have been observed in conventionally-treated cereal fields compared to the respective frequencies in organic fields [30,31]. Such differences had been attributed to changes in the saprophytic microflora of the conventionally treated plants or differences in the canopy microclimate. However, no data have been provided on the fungicide resistance status of the fungal populations from either conventional or organic farming systems that could explain the observed differences.

Monitoring data showed that within the *A. uvarum* population sampled, which was the predominant species in the conventionally treated vineyards, relatively high frequencies of resistance to the respiration inhibitors fluxapyroxad (SDHIs) and pyraclostrobin (QoIs) occurred. Interestingly, almost all of the *A. uvarum* isolates exhibiting high resistance levels to fluxapyroxad or pyraclostrobin originated from conventionally treated vineyards. These two fungicide classes are currently the most commonly used in vineyards against the main grape diseases; thus, black aspergilli populations are heavily exposed to selection pressure even if they are not always the target of these fungicide applications. Similarly, the few isolates of *A. uvarum* or *A. tubingensis* that were found to be highly resistant to fludioxonil were all obtained from the conventionally-treated fields. However, marked differences were observed between the two species in the frequencies of isolates with low resistance levels to this fungicide. The frequency of the *A. uvarum* isolates originating from conventionally-treated vineyards with low resistance levels to fludioxonil was 50.7, while the respective frequency value for the *A. tubingensis* isolates was only 15.7%. Thus, the observed resistance profile of *A. uvarum* may explain its predominance in the vineyards of a conventional farming system where target-specific fungicides are heavily used to combat powdery mildew, downy mildew, or gray mold diseases. Most likely, the selected fungicide-resistant strains of *A. uvarum* escape the fungicidal activity of the applied products and increase in frequency compared to other black aspergilli species that are fungicide sensitive. The above data provide evidence of the role of fungicide applications in shaping fungal populations. Fungicide applications are recognized in modern agriculture as a major agent of genetic drift events within fungal populations, contributing to pathogen evolution by reducing the population size, altering their composition, and shaping their diversity [32,33]. In addition to the effect of fungicide applications in conventionally-treated vineyards and the emergence of fungicide resistant strains discovered in the current study, other factors such as differences in the core microbiome of the grape bunches may affect the presence and composition of black aspergilli and contribute to differences between conventional and organic vineyards, which merit further investigation.

The H270Υ mutation that was detected in several *A. uvarum* isolates mainly originated from the conventionally treated vineyards has been previously reported in several other plant pathogenic fungi such as *Botrytis cinerea*, *Zymoseptoria tritici*, *Alternaria alternata*, and others [34,35], while it has also been reported in the closely related *Aspergillus oryzae* used in the fermentation industry [36]. Similarly, the presence of H270Y or H270R mutations was reported in a limited number of *A. fumigatus* strains collected from agricultural environments [37,38], while the homologue mutation H249Y has been reported in *A. flavus* strains with laboratory-induced resistance to SDHIs [39]. The current study represents the first report of resistance development to SDHIs in a plant pathogenic *Aspergillus* spp. The identified H270Y *sdhB* mutation has been correlated with moderate to high levels of resistance to several SDHIs such as the pyrazole carboxamides fluxapyroxad and penthiopyrad, or the pyridine carboximide boscalid in *B. cinerea*, *Corynespora cassiicola*, or *Didymella bryoniae*. In contrast, the same mutation has been found to confer hypersensitivity to other SDHI molecules such as the benzamide molecules fluopyram and benodanil or the thiophene amide isofetamid [40,41]. Our results are in line with the above-mentioned studies since we observed a similar pattern of sensitivity toward several of the SDHIs tested in the H270Y mutants of *A. uvarum*. Interestingly, a double mutation (H65Q/P66S) located in *sdhD* was observed in a limited number of *A. uvarum* isolates showing moderate levels of resistance to fluxapyroxad. As far as we know, such mutations or their homologs have not been reported in any other fungal species yet. However, several other mutations in *sdhD* have been reported in several plant pathogenic species conferring low to moderate resistance to SDHIs [42]. Cross-resistance studies showed that the isolates possessing this double substitution in *sdhD* were of low to moderate resistance in all five (5) SDHI molecules tested. Interestingly, a small fraction of the resistant isolates possessed both the H270Y and the H65Q/P66S mutations. However, measurements of the sensitivity of these isolates to a set of several SDHIs revealed that their sensitivity phenotype was similar to that of H270Y mutants. This is most probably related to the fact that *sdhB* mutations exhibit a stronger effect on the resistance levels of the mutated strains compared to those caused by *sdhC/D* mutations [36]. In contrast to *A. uvarum*, *A. tubingensis* isolates with only low resistance levels to fluxapyroxad were detected, but the sequence of *sdhB*, *sdhC*, and *sdhD* in these isolates did not reveal any point mutation compared to the sequence of the sensitive strains. These low levels of resistance to SDHIs may be explained by increased fungicide efflux due to the hyperactivity of efflux pumps, but experimental evidence is required. The role of efflux transporters as a resistance mechanism has been extensively studied in *A. fumigatus* due to its medical value or in *Aspergillus* spp. used as model species, but no information is known for agriculturally important *Aspergillus* spp. [43,44,45]. In A. carbonarious, no mutation was identified in the *sdhB/C/D* subunits, but this fungal species is expected to be insensitive to SDHIs. A similar finding has been reported by Kalampokis et al. [46], suggesting that *A. carbonarious* isolates are insensitive to the SDHI boscalid.

Resistance to QoIs was only found in a certain number of the *A. uvarum* isolates, whereas all three (3) remaining black aspergilli species were found to be highly sensitive to pyraclostrobin. The sequencing of *cytb* in the resistant isolates revealed that all of them possessed a glycine replacement by alanine at codon 143 (G143A). This mutation is by far the most common mutation associated with resistance to QoIs in plant-pathogenic fungi and has been reported in more than 30 different plant-pathogenic fungal species [47]. The G143A mutation confers very high levels of resistance to QoIs, and leads to the complete loss of efficacy of QoIs against their resistant target species, while in most cases, isolates possessing this mutation do not suffer any fitness penalty. Interestingly, although almost half of the *A. tubingensis* included in this study originated from conventionally-treated vineyards, none of them were found to be resistant to QoIs. In several plant pathogens, the absence of resistance to QoIs associated with the G143A mutation has been associated with variations in the arrangements of exons and introns in *cyt*b. In detail, the occurrence of an intron directly after codon 143 might have a significant impact on the splicing process, resulting in cytochrome b deficiency, and organisms with these alterations would not survive [48,49]. In addition to G143A, the replacement of phenylalanine by leucine at codon 129 (F129L) or the replacement of glycine by arginine at position 137 (G137R) have been reported in a limited number of pathogenic plant species such as *Alternaria solani*, *Passalora fulva*, or *Pyrenophora tritici-repentis* [50,51], but both are associated with relatively low levels of resistance to QoIs. Resistance to QoIs in *A. uvarum* strains, isolated from grapes, has very recently been reported in the mid-Atlantic U.S. [24]. Interestingly, in this case, resistance was associated with the F129L mutation, conferring moderate levels of resistance to QoIs as expected. Thus, the combination of our data with the data reported by Cosseboom and Hu [22] suggests that *A. uvarum* belongs to the short list of plant pathogens in which the presence of both F129L and G143A mutations have been reported including *Plasmopara viticola*, *Magnaporthe grisea*, and *Zymospetoria tritici* [52,53]. In *Aspergillus flavus* isolates from peanut seeds, both the F129L and G143A mutations were identified in different strains of the population [54].

Resistance to DMIs was not found to be of major importance in the present samples. High resistance to tebuconazole was observed only in a limited number of *A. uvarum* isolates. However, in all of these isolates, no mutation was observed in the target gene *Cyp51*. This is in agreement with previous reports suggesting that *Cyp51* mutations do not play an important role in *Aspergillus* section *Nigri* resistance to azole fungicides as occurs with *A. fumigatus* clinical isolates [55]. Indeed, in *A. fumigatus*, several *Cyp51* mutations or combinations of *Cyp51* mutations with the *Cyp51* promoter tandem repeats act as transcription enhancers and have been identified as the most common resistance mechanism to DMIs [22,37,56]. Most probably, other resistance mechanisms such as increased expression of the target gene or increased fungicide efflux may account for the observed phenotype in the *A. uvarum* isolates with resistance to tebuconazole [57,58]. Further studies are required to fully elucidate the precise mechanism of resistance to DMIs in these isolates. In addition to this, continuous monitoring of black aspergilli sensitivity to DMIs is required, taking into account that the exposure of *Aspergillus* strains to DMIs in agricultural environments increases the risk for the selection of resistance to azoles in strains associated with invasive aspergillosis in human patients [59].

Similar to DMIs, high levels of resistance to the phenylpyrrole fungicide fludioxonil were observed in a limited number of *A. uvarum* and *A. tubingensis* isolates, while isolates of *A. carbonarius* or *A. niger* were highly sensitive to this active ingredient. Interestingly, within the *A. uvarum* population tested, 43.1% of the isolates were found to be lowly resistant to fludioxonil, while in the *A. tubingensis* subpopulation, only 14.5% of the isolates were lowly resistant to fludioxonil. Field resistance to this fungicide class is associated with target site modifications in the histidine kinase Os gene or Os-like genes [60]. However, reports of resistance development to fludioxonil due to target site modifications in the field are very rare and have been described in a limited number of phytopathogenic fungi that mostly include several *Alternaria* spp. [61]. This is most probably due to fitness defects in field isolates that possess these target site alterations, as has been reported in several fungal species [62,63]. In contrast to the rarity of fludioxonil resistance due to target site modifications, several cases of low or moderate levels of resistance to fludioxonil have been reported in fungal species such as *Botrytis cinerea* or *Penicillium expansum* due to the overexpression of ABC or MFS transporters [64,65]. In the current study, due to the low frequencies of highly resistant isolates to fludioxonil, we did not emphasize the molecular mechanisms of resistance to the fungicide class. However, further research aiming to determine the precise mechanism of resistance to fludioxonil should elucidate this.

## 5. Conclusions

The results of this study provide evidence on the effect of fungicide resistance development on the black aspergilli incidence on grapes and population structure at the species level. Within the population tested, higher resistance frequencies were observed in *A. uvarum* isolates originating mostly from conventionally treated vineyards. In this fungal species, moderate frequencies of resistance were detected to respiration inhibitors such as QoIs and SDHIs. Active ingredients belonging to these two fungicide classes are commonly used in conventionally-treated vineyards to combat several foliar or bunch diseases, and black aspergilli populations are exposed to them mostly as off-target pathogens. Fungicide applications in the conventionally-treated vineyards induce selection pressure and change the composition of black aspergilli within the vineyards. The sequencing of the target genes of QoIs and SDHIs in the resistant *A. uvarum* isolates showed that the resistance to QoIs was associated with the presence of the G143A mutation in *cytb*, while the SDHI-resistant isolates possessed the H270Y mutation in *sdhB* or a double substitution of H65Q/P66S in *sdhD*. In addition to resistance to QoIs and SDHIs, low resistance levels were detected against the DMI tebuconazole in both *A. uvarum* and *A. tubingensis*, but after sequencing of the target genes *Cyp51A* and *Cyp51B*, no mutation was identified, leading to the conclusion that the observed resistance phenotype was most probably associated with the increased activity of efflux transporters. Such findings will help design appropriate strategies to manage Aspergillus bunch rot in vineyards while underlining the risk of the selection of black aspergilli-resistant strains with clinical importance since this selection is based on resistance developed in agricultural environments and not on long-term exposure to fungicides during medical therapy.

## Figures and Tables

**Table 1:** Designed primer pairs for the amplification and the sequencing of PCR products for the *sdhB*, *sdhC*, *sdhD*, *Cyp51A*, *Cyp51B*, and *cytb* subunits of the *Aspergillus uvarum* and *A. tubingensis* isolates.

*Aspergillus* Species	Primer ID	Target	Forward (5’→3’)	Reverse (5’→3’)	Amplicon Size (bp)	Annealing Temperature (° C)
*Aspergillus uvarum*	sdhB_uva_Fw/Rv	*sdh*B	CAGTGCCTTCAGGGTCTATCC	CGCCCTTTATTTTGCTGCCA	1488	65
	sdhC_uva_Fw/Rv	*sdh*C	TCTTTCCTGGTCGACCACGA	TGACTGGCTATGGAATATATATTGCA	971	60
	sdhD_uva_Fw/Rv	*sdh*D	TCGAACCGCGATCACCAATC	ACACTCATTTTATGCTCCAGGGT	1217	65
	Cyp51a_uva_Fw/Rv	*Cyp51*A	GCGATGTAAACAGTACGGCG	GAGTTGGGGCAGTGCTACAC	1782	65
	Cyp51b_uva_Fw/Rv	*Cyp51*B	TCTCAGCCACAATCACCCTG	GTATATATACATTAGCTTCTCAGCAGC	2136	65
	Cytb_asp2_Fw/Rv	*Cytochrome* b	TGTAAATAATGGTTGATTAGTACGTT	TGGTCTGAATTGTACTCCTCT	752	60
*Aspergillus tubingensis*	SdhB_tub_Fw/Rv	*sdh*B	TCGCCATCTGGTAGATCCCT	TCCGACATGAAAGACCTCGG	1232	65
	SdhC_tub_Fw/Rv	*sdh*C	GGTTTCCCCGGCTGCTGTTG	ACAGGAAAGCAAGAATGAGAGCGC	584	65
	SdhD_tub_Fw/Rv	*sdh*D	GACCAATCAGAGGCACGACA	AAGTACATCTTTTATAGCTCGATGAGC	1235	65
	Cyp51a_tub_Fw/Rv	*Cyp51*A	GCCTCCTTCCGTTGCTAACA	CGTGGAGAGTCCCGGAGATA	1788	65
	Cyp51b_tub_Fw/Rv	*Cyp51*B	CGGTCATTCTGTTTCCGCTG	TGCAGTAACAGAGGCAGGTC	1813	65

**Table 2:** Sensitivity measurements of *Aspergillus* section *Nigri* species from grapes to fluxapyroxad, tebuconazole, pyraclostrobin, and fludioxonil.

Species	Fungicide	Phenotype ^a^	Number of Isolates	Range of EC_50_ Values ^b^	Mean EC_50_	Resistance Factor ^c^
*A. uvarum*	fluxapyroxad	S	52	0.02 to 0.29	0.15	0.13 to 1.93
		LR	33	0.3 to 4.33	0.93	2 to 29
		HR	17	>10	>10	>67
	tebuconazole	S	96	0.01 to 0.18	0.1	0.1 to 1.83
		LR	nd ^d^	nd	nd	nd
		HR	6	>1	>1	>10
	pyraclostrobin	S	86	0.0008 to 0.078	0.04	0 to 1.96
		LR	nd	nd	nd	nd
		HR	16	>100	>100	>2500
	fludioxonil	S	56	0.004 to 0.019	0.01	0.3 to 1.92
		LR	44	0.02 to 0.13	0.05	2 to 13.3
		HR	2	>1	>1	>100
*A. tubingensis*	fluxapyroxad	S	120	0.00006 to 0.097	0.05	0 to 1.95
		LR	31	0.10 to 3.52	0.34	2 to 70.4
		HR	nd	nd	nd	nd
	tebuconazole	S	93	0.01 to 0.89	0.46	0 to 1.93
		LR	58	0.93 to 2.85	1.35	2 to 6.19
		HR	nd	nd	nd	nd
	pyraclostrobin	S	151	0.0004 to 0.026	0.02	0 to 1.3
		LR	nd	nd	nd	nd
		HR	nd	nd	nd	nd
	fludioxonil	S	129	0.002 to 0.038	0.02	0 to 1.9
		LR	18	0.042 to 0.08	0.05	2.1 to 4
		HR	4	>1	>1	>50
*A. niger*	fluxapyroxad	S	19	0.03 to 0.06	0.04	0.7 to 1.5
		LR	nd	nd	nd	nd
		HR	nd	nd	nd	nd
	tebuconazole	S	11	0.1 to 0.45	0.25	0.4 to 1.8
		LR	8	0.52 to 1.32	0.77	2.1 to 5.3
		HR	nd	nd	nd	nd
	pyraclostrobin	S	19	0.0001 to 0.008	0.001	0 to 0.8
		LR	nd	nd	nd	nd
		HR	nd	nd	nd	nd
	fludioxonil	S	19	0.0143 to 0.038	0.02	0.7 to 1.9
		LR	nd	nd	nd	nd
		HR	nd	nd	nd	nd
*A. carbonarius*	fluxapyroxad	S	nd	nd	nd	nd
		LR	nd	nd	nd	nd
		HR	22	>20	>20	N/A ^e^
	tebuconazole	S	14	0.05 to 0.44	0.23	0.2 to 1.9
		LR	8	0.51 to 1.18	0.71	2.2 to 5.1
		HR	nd	nd	nd	nd
	pyraclostrobin	S	22	0.00006 to 0.024	0.02	0 to 1.2
		LR	nd	nd	nd	nd
		HR	nd	nd	nd	nd
	fludioxonil	S	20	0.0061 to 0.032	0.02	0.3 to 1.6
		LR	2	0.04 to 0.09	0.09	2 to 4.5
		HR	nd	nd	nd	nd

^a^ Phenotype: S: Sensitive, LR: Low resistance, HR: High resistance; ^b^ EC50 values in mg L-1; ^c^ Resistance Factor values were determined by dividing the EC50 value of each isolate by the mean EC50 value of the most sensitive isolates; ^d^ nd: non detected; ^e^ N/A: not applicable.

**Table 3:** EC_50_ and resistance factor (RF) values to several SDHI fungicides of sensitive and resistant *Aspergillus uvarum* isolates, possessing the H270Y mutation in the *sdhB* gene and/or the H65Q/P66S mutations in the *sdhD* gene.

Isolate	Phenotype	Fluxapyroxad		Boscalid		Penthiopyrad		Isofetamid		Fluopyram	
		EC50 ^a^	RF ^b^	EC50	RF	EC50	RF	EC50	RF	EC50	RF
A66	Sensitive	0.0051	1.7	0.0045	0.9	0.0345	1.8	0.0077	1.6	0.005	0.8
A147	Sensitive	0.0045	1.5	0.0034	0.7	0.0048	0.3	0.0039	0.8	0.008	1.5
A88	Sensitive	0.0012	0.4	0.0071	1.4	0.0178	0.9	0.0031	0.6	0.005	0.8
A137	H270Y	0.2424	80.8	1.3150	263	3.3080	174	0.0004	0.1	0.001	0.1
A127	H270Y	0.6251	208	1.4900	298	2.4160	127	0.0035	0.7	0.002	0.3
B7	H270Y	1.9920	664	0.9796	195	2.6590	140	0.0023	0.5	0.004	0.6
B9	H270Y	0.1517	50.5	1.0370	207	0.3942	20.7	0.0037	0.7	0.003	0.5
B14	H65Q/P66S	0.0150	5.0	0.2205	44.1	0.0632	3.3	0.0261	5.3	0.098	16.3
A67	H65Q/P66S	0.0168	5.6	0.0605	12.1	0.0527	2.7	0.0183	3.7	0.094	15.6
A121	H65Q/P66S	0.0461	15.3	0.1290	25.8	0.0842	4.4	0.1381	28.1	0.332	55.3
A69	H65Q/P66S	0.0196	6.5	0.0606	12.1	0.0763	4.0	0.0466	9.5	0.085	14.2
B144	H65Q/P66S	0.0209	6.9	0.0591	11.8	0.1084	5.7	0.0682	13.9	0.193	32.1
B12	H270Y+H65Q+P66S	0.6217	207	0.8671	173	1.4010	73.7	0.0019	0.4	0.004	0.6
B10	H270Y+H65Q+P66S	0.2446	81.5	0.8866	177	0.3996	21.0	0.0016	0.3	0.005	0.8
B11	H270Y+H65Q+P66S	0.1366	45.5	0.8942	178	0.4867	25.6	0.0009	0.1	0.003	0.5

^a^ EC50 values in μg ml-1; ^b^ RF values were calculated by dividing the isolate EC50 value by the mean EC50 values for the non-mutated isolates of 0.003, 0.005, 0.019, 0.0049 and 0.006 μg ml-1 for fluxapyroxad, boscalid, penthiopyrad, isofetamid and fluopyram, respectively.

## Data Availability

All experimental data are provided in the manuscript and in the supplementary files.

## Supplementary Materials

The following supporting information can be downloaded at: https://www.mdpi.com/article/10.3390/jof9020221/s1, Table S1: Number of *Aspergillus* section *Nigri* isolates collected from several conventional or organic vineyards in Greece and used for fungicide sensitivity measurements; Table S2: Range of fungicide concentrations (mg L^−1^) used to determine the sensitivity profile of several *Aspergillus* section *Nigri* isolates to fluxapyroxad, tebuconazole, fludioxonil, and pyraclostrobin; Table S3: Published sequences of the *sdhB*, *sdhC*, *sdhD*, *cytb*, *Cyp51A*, and *Cyp51B* genes used to design the respective primer pairs for the *Aspergillus uvarum* and *A. tubingensis* resistant isolates; Table S4: Accession numbers of the *Aspergillus uvarum* sequences of sensitive and resistant isolates possessing the H270Y, H65Q/P66S, and G143A mutations in *the sdhB*, *sdhD*, and *cytb* encoding genes, respectively. The sequences were submitted to the publicly available database of the National Center of Biotechnology and Information (NCBI).
