# Supplementary material for: Resistance of Black Aspergilli Species from Grape Vineyards to SDHI, QoI, DMI, and Phenylpyrrole Fungicides
Source: J Fungi (Basel). 2023 Feb 7;9(2):221. doi: 10.3390/jof9020221 (PMC9961879; doi:10.3390/jof9020221)
Supplement: Supplementary file 1 [file jof-09-00221-s001.zip › jof-2207997-supplementary.pdf]

Journal of Fungi

Table S1. Number of *Aspergillus* section *Nigri* isolates collected from several conventional or organic vineyards in Greece and used for fungicide sensitivity measurements.

Aspergillus Species	Total	Farming System	
		Conventional	Organic
A. uvarum	102	79	23
A. tubingensis	151	76	75
A. niger	19	12	7
A. carbonarius	22	13	9

Table S2. Range of fungicide concentrations (mg L^{-1}) used to determine the sensitivity profile of several *Aspergillus* section *Nigri* isolates to fluxapyroxad, tebuconazole, fludioxonil, and pyraclostrobin.

Aspergillus spp.	Fungicide			
	fluxapyroxad	tebuconazole	fludioxonil	pyraclostrobin
A. uvarum	0, 0.05, 0.1, 0.5, 1, 2.5, 5, 10	0, 0.001, 0.005, 0.01, 0.05, 0.1, 0.5, 1	0, 0.001, 0.005, 0.01, 0.05, 0.1, 0.5, 1	0, 0.001, 0.005, 0.01, 0.05, 0.1, 0.5, 1, 5, 10, 50, 100
A. tubingensis	0, 0.1, 0.5, 1, 2.5, 5, 10	0, 0.05, 0.1, 0.5, 1, 2.5, 5, 10, 20	0, 0.01, 0.05, 0.1, 0.5, 1, 5	0, 0.001, 0.005, 0.01, 0.05, 0.1, 0.5, 1, 5, 10, 50, 100
A. carbonarius	0, 0.05, 0.1, 0.5, 1, 2.5, 5, 10, 20	0, 0.05, 0.1, 0.5, 1, 2.5, 5, 10, 20	0, 0.001, 0.005, 0.01, 0.05, 0.1, 0.5, 1	0, 0.001, 0.005, 0.01, 0.05, 0.1, 0.5, 1, 5, 10, 50, 100
A. niger	0, 0.05, 0.1, 0.5, 1, 2.5, 5, 10	0, 0.05, 0.1, 0.5, 1, 2.5, 5, 10, 20	0, 0.001, 0.005, 0.01, 0.05, 0.1, 0.5, 1	0, 0.001, 0.005, 0.01, 0.05, 0.1, 0.5, 1, 5, 10, 50, 100

Table S3. Published sequences of *sdhB, sdhC, sdhD, cytb, Cyp51A,* and *Cyp51B* genes used to design the respective primer pairs for *Aspergillus uvarum* and *A. tubingensis* resistant isolates.

Aspergillus Species	Target Gene	Reference Sequencing	Full Genome (Map2Ref)
Aspergillus uvarum	*sdhB*	1. *A. japonicus* (XM_025671997.1)<br>2. *A. uvarum* (XM_025631809)	*Aspergillus uvarum (CBS 121591)*<br>*1.* GenBank assembly accession: GCA_003184745.1<br>*2.* BioProject: PRJNA235077
	sdhC	1. *A. uvarum* (XM_025638107.1)<br>2. *A. japonicus* (XM_025672799.1)<br>3. *A. niger* (XM_001391363.2)	
	sdhD	1. *A. uvarum* (XM_025633517.1)<br>2. *A. japonicus* (XM_025672565.1)	
	Cyp51A	1. *A. uvarum* (XM_025636060.1)<br>2. *A. japonicus* (XM_025672537.1)	
	Cyp51B	1. *A. uvarum* (XM_025633329.1)<br>2. *A. japonicus* (XM_025668681.1)	
	Cytochrome b	1. *A. niger* (DQ178141.1)<br>2. *A. japonicus* (MN960692.1)<br>3. *A. tubingensis* (DQ217399.1)	
Aspergillus tubingensis	*sdhB*	1. *A. tubingensis* (XM_035496087.1)<br>2. *A. niger* (XM_025598493.1)<br>3. *A. flavus* (MT226811.1)	*Aspergillus tubingensis* (CBS 134.48)

	sdhC	1. *A. tubingensis* (XM_035502954.1)<br>2. *A. costaricaensis* (XM_025682433.1)<br>3. *A. niger* (XM_001391363.2)	1. GenBank assembly accession: GCA_001890745<br>2. BioProject: PRJNA235077
	sdhD	1. *A. neoniger* (XM_025622088.1)<br>2. *A. tubingensis* (XM_035499107.1)	
	Cyp51A	1. *A. tubingensis* (XM_035501642.1)<br>2. *A. tubingensis* (JF450912.1)	
	Cyp51B	1. *A. tubingensis* (XM_035504231.1)<br>2. *A. costaticaensis* (XM_025685518.1)	

Table S4. Accession numbers of *Aspergillus uvarum* sequences of sensitive and resistant isolates possessing the H270Y, H65Q/P66S, and G143A mutations in the *sdhB, sdhD,* and *cytb* encoding genes, respectively. The sequences were submitted to the publicly available database of the National Center of Biotechnology and Information (NCBI).

Isolate	Target gene	Phenotype	Mutation	Accession Number
B85	*sdhB*	Sensitive	-	OP142304
A112	*sdhB*	Sensitive	-	OP142305
A97	*sdhB*	Resistant	H270Y	OP142306
B12	*sdhB*	Resistant	H270Y	OP142307
B11	*sdhB*	Resistant	H270Y	OP142308
B5	*sdhD*	Sensitive	-	OP142309
B138	*sdhD*	Sensitive	-	OP142310
A67	*sdhD*	Resistant	H65G/P66S	OP142311
A69	*sdhD*	Resistant	H65G/P66S	OP142312
A121	*sdhD*	Resistant	H65G/P66S	OP142313
B142	*cyt b*	Sensitive	-	OP142314
A83	*cyt b*	Resistant	G143A	OP142315
A84	*cyt b*	Resistant	G143A	OP142316
A134	*cyt b*	Resistant	G143A	OP142317
